# Safety evaluation of matrine and its impact as a feed additive on the production performance of piglets

**DOI:** 10.3389/fvets.2025.1605448

**Published:** 2025-07-08

**Authors:** Zhigang Cao, Yalin Wu, Xiangming Cong, Abdul Haseeb, Panpan Sun, Hua Zhang, Kuohai Fan, Wei Yin, Huizhen Yang, Zhenbiao Zhang, Jia Zhong, Jianzhong Wang, Yaogui Sun, Hongquan Li, Na Sun

**Affiliations:** ^1^Shanxi Key Laboratory for Modernization of TCVM, College of Veterinary Medicine, Shanxi Agricultural University, Taigu, Shanxi, China; ^2^Manhua Medical Technology Co., Ltd, Weihai, Shandong, China; ^3^Laboratory Animal Center, Shanxi Agricultural University, Taigu, Shanxi, China

**Keywords:** matrine, weaned piglets, feed additives, growth performance, *Gemmiger formicilis*, thiamine

## Abstract

**Introduction:**

Previous studies have suggested that matrine may improve animal production performance, but its role and underlying mechanisms remain unclear.

**Methods:**

Following the determination of the LD_50_ of matrine in ICR mice, the long-term toxic effects of matrine on SD-weaned rats were evaluated. 0.375, 0.75, 1.5, and 3 mg/kg matrine were added to the feed of weaned piglets, respectively. The feed intake and body weight of piglets were recorded to evaluate the growth-promoting effect of matrine. The feces and blood of weaned piglets were collected to explore the mechanism of matrine improving the growth performance of piglets.

**Results:**

Our findings imply that the LD_50_ of matrine in mice was 202.54 mg/kg, and matrine did not cause any hazardous effects when administered to rats within the range of 24.5–50 mg/kg for 180 days. Furthermore, supplementation of 0.375, 0.75, and 1.5 mg/kg matrine can increase ADG (average daily weight gain), and ADFI (average daily feed intake), and decrease the FCR (food conversion rate) of piglets. Additionally, 0.375 and 0.75 mg/kg matrine could increase the positive rate of porcine circovirus type 2 (PCV2) vaccine antibody in serum of piglets. We analyzed the correlation between intestinal flora, fecal metabolites, and growth performance through Mothur software and found that the impact of matrine on ADG, ADFI, and FCR might be associated to *Gemmiger formicilis* and thiamine.

**Conclusion:**

These findings revealed that matrine can improve the growth performance of weaned piglets by increasing the abundance of *Gemmiger formicilis* and thiamine content in feces.

## 1 Introduction

China dominates the world in pork production and consumption. According to the latest data from the National Bureau of Statistics, 726.62 million pigs were slaughtered in China in 2023, an increase of 26.68 million compared with 2022, an increase of 3.8%. Pork production was 57.94 million tons, an increase of 2.53 million tons compared with 2022, an increase of 4.6%. Therefore, China’s substantial demand for pork positions it as the top consumer and producer of pork globally. China has yet to become a leading pork-producing country and continues to face enormous challenges in disease prevention and control, growth performance improvement, and production efficiency enhancement (1, 2). Affected by pandemics such as PCV2 and higher breeding costs, pig breeding has been in a state of financial decline at present. According to the latest statistics from the Ministry of Agriculture and Rural Affairs of the People’s Republic of China, in 2023, pig farmers experienced an average loss of 76 yuan per pig, marking the first year of total annual losses since 2014. Therefore, amidst the prevalence of pathogens such as African swine fever virus (ASFV) (3), porcine reproductive and respiratory syndrome virus (PRRSV) (4), and PCV2 (5), strengthen the immune function and growth performance of piglets to enhance farming profitability has become an urgent issue in pig farming.

In 1994, the Ministry of Agriculture and Rural Affairs of the People’s Republic of China issued the “List of Permitted Feed Additives,” including antibiotics as feed additives. The use of antibiotics significantly enhanced the economic benefits of animal husbandry. However, adverse effects such as drug residues and bacterial resistance caused by the misuse of antibiotics pose a significant threat to human safety (6). Therefore, in 2019, the Ministry of Agriculture and Rural Affairs of the People’s Republic of China released Announcement No. 194, which explicitly banned and prohibited all growth-promoting feed additives except traditional Chinese medicine. Against the backdrop of “antibiotic-free feed and reduced resistance in farming,” traditional Chinese veterinary medicine feed additives that improve livestock growth performance and enhance immune function have become research hotspots. Currently, only a limited number of new veterinary drug feed additives have been approved in China, including Boluohui San, Fructus Ligustri Lucidi, Kingfoo Herbs, Callicarpanudiflora Power, Chlorogenic Acid, Silymarin, Red Clover Extract, Mosla Chinensis Maxim Extractives, and Marigold Extract. Among these, Boluohui San (commercially known as Sangrovit) is a powder processed from the extract of *Macleaya cordata* (7). Its primary active components are sanguinarine and chelerythrine (8). Since the main active components of Boluohui San, sanguinarine and chelerythrine, like matrine, are plant-derived alkaloids (nitrogen-containing organic compounds) and have demonstrated that dietary supplementation with 50 mg/kg Boluohui San significantly enhances growth performance in weaned piglets (9), we selected Boluohui San as the positive control in our experiments. As one of China’s first approved Class II veterinary drugs and feed additives, the Announcement No. 2374 issued by the Ministry of Agriculture and Rural Affairs of the People’s Republic of China also indicates that dietary supplementation with 20 ~ 50 mg/kg Boluohui San can enhance the growth performance of pigs. Nevertheless, the repertoire of approved traditional Chinese veterinary feed additives remains limited, underscoring an ongoing need for novel developments in this field.

Matrine is an alkaloid extracted from *Sophora flavescens*. A large number of studies have shown that matrine has anti-inflammatory (10), anti-oxidation (11), anti-tumor (12), and other pharmacological effects. Our previous studies have found that matrine has a good inhibitory effect on PCV2 (13), PRRSV (14), PRRSV/PCV2 co-infection (15), encephalomyocarditis virus (EMCV) (16), and other viral infections. Through 16S rDNA sequencing and fecal microbiota transplantation, we found that matrine can modulate the gut microbiota structure in Kunming mice. Specifically, it increases the abundance of beneficial bacteria such as *Lactobacillus* and *Lactobacillus acidophilus* in the mouse gut, thereby exerting an inhibitory effect on PCV2 (17). Studies have shown that the effects of feed additives such as Boluohui San (18) and Chlorogenic Acid (19, 20) on promoting growth and improving immune function are related to the regulation of intestinal flora such as *Lactobacillus*. Huang et al. found that the administration of *Lactobacillus* to piglets can regulate the changes of intestinal flora structure and metabolic level of piglets, thereby improving the growth performance and immunity of piglets during lactation (21). Based on the improved effect of matrine on intestinal flora such as *Lactobacillus acidophilus*, it is suggested that matrine may also have the effect of improving animal growth performance and immune function and is expected to be developed and registered as a feed additive.

Therefore, the safety of matrine was evaluated by acute toxicity test in mice and long-term toxicity test in rats. The 28-day-old weaned piglets were selected to design the effect of matrine on the growth performance and immune function of weaned piglets and to evaluate the clinical effectiveness of matrine as a feed additive. In order to analyze the mechanism of the effect of matrine on the growth performance of weaned piglets, metagenomics was used to analyze the effect of matrine on the intestinal flora structure of weaned piglets, and non-targeted metabolomics was used to detect the effect of matrine on fecal metabolites of weaned piglets. To provide production and theoretical basis for the development of matrine as a traditional Chinese veterinary medicine feed additive.

## 2 Materials and methods

### 2.1 Animals

SPF ICR mice, weighing between 18 and 22 g with equal ratio of males and females, were obtained from Beijing Sipeifu Biotechnology Co., Ltd. (Quality Certificate Number: 110324230103270157). SPF SD rats, half male and half female, 28 days old, were purchased from Beijing Sipeifu Biotechnology Co., Ltd. (Quality Certificate Number: 110324230103269824). All mice and rats were raised in the Experimental Animal Management Center of Shanxi Agricultural University. A total of 1,200 28-day-old healthy weaned piglets weighing about 8.5 ± 2 kg were provided by Huanshan Group Co., Ltd.

### 2.2 Piglet management and feed formulation

During the trial, piglets were provided with ad libitum access to feed and water. The pens were maintained under dry and hygienic conditions, with four daily feedings and once-daily manure removal. Appetite, diarrhea incidence, and mental/behavioral status of the weaned piglets were observed and recorded regularly. All other management practices followed routine protocols for piglet rearing, including epidemic prevention, disinfection, and vaccination according to the farm’s standard procedures. The basal diet formulation for piglets is presented in Table 1. To prepare the matrine-supplemented diets, matrine was first thoroughly mixed with potato starch at a ratio of 1:1,000 (w/w). This premix was then blended into the basal diet to achieve final matrine concentrations of 0.375, 0.75, 1.5, and 3 mg/kg feed.

**Table 1:** Ingredient composition and nutritional values of basal diets (air-dried basis).

Items	Content	Nutrient	Content
Corn	59.15	Digestible energy, MJ/kg	14.85
Soybean meal	16.0	Crude protein, %	18.88
Expanded soybean	13.0	Standard total gastrointestinal digestibility phosphorus, %	0.36
Fish meal	2.80	Calcium, %	0.73
Whey	3.00	Lysine, %	1.23
Soybean oil	2.00	Threonine, %	0.74
Limestone	0.80	Methionine, %	0.47
Calcium hydrogen phosphate	1.00	Tryptophan, %	0.20
NaCl	0.25	Methionine + Cysteine, %	0.69
Premix	2.00		
Total	100.00		

### 2.3 Medicines

Matrine (Batch No: ZLSC2018032020), purity 98.7%, purchased from Nanjing Zelang Biotechnology Co., Ltd. Boluohui San (Batch No: 180415250), specification: 100 g: 3.75 g, gifted by Professor Zeng Jianguo of Hunan Agricultural University.

### 2.4 Acute toxicity test of matrine

The acute toxicity test of matrine was designed according to the “Guidelines for Acute Toxicity Test of Veterinary Drugs (LD_50_ Determination).” After weighing, the mice were intragastrically administered at a dosage of 0.2 mL/10 g. The 0/4 lethal dose (a) and 4/4 lethal dose (b) of matrine were determined to be 50 mg/kg and 300 mg/kg, respectively. According to the ratio of b and a, the number of formal test groups (N) was determined to be 6 groups. Based on the formula, the dose ratio between two consecutive groups was 1: 0.7, calculated as (*r* = lg−1 [(lg (b) −lg (a))/(N−1)]), where a is the 0/4 lethal dose, b is the 4/4 lethal dose, and N represents the number of dose groups. Therefore, the dosages of matrine in the six dose groups were 300, 210, 147, 102.9, 72.03, and 50.42 mg/kg, respectively. Each group consisted of 10 rats, with an equal distribution of male and female species. After intragastric administration, the mice were monitored for a duration of 7 days, during which the symptoms and death time of the mice were recorded. LD_50_ (LD_50_ = lg-1 [X_m_-i (∑ p-0.5)]) was calculated by the formula. The 95% confidence interval was calculated as, lg-1 (lgLD_50_ ± 1.96 × i$\sqrt{\sum (\mathrm{pq}/n)}$), where *X_m_* is the logarithm of the maximum dose; *i* is the group distance; *p* is the mortality of each group; *q* is the survival rate of each group; *∑ p* is the sum of the mortality of each group; *n* is the number of animals in each group.

### 2.5 Long-term toxicity test of matrine

The long-term toxicity test of matrine was designed in accordance with the “Technical Guidelines for the Safety and Effectiveness of Growth-Promoting Veterinary Chinese Medicines” and “Guidelines for 30- and 90-days feeding trials of veterinary drugs.” A cohort of 80 SPF SD-weaned rats (4 weeks), both male and female in equal proportion were randomly allocated into four groups, each consisting of 20 rats. These groups were designated as the negative control group (NC), low dose group (L), medium dose group (M), and high dose group (H). The highest dose was 25% of the LD_50_ (Median lethal dose) in the acute toxicity test. In order to ensure that the minimum dose was 3 times higher than the target animal intake, the doses of the low, medium, and high groups were determined to be 24.5, 35, and 50 mg/kg, respectively. The rats were intragastrically administered with 1 mL/100 g for 180 days. The general behavior, poisoning, and mortality rate of the animals were observed and recorded every day. At the 90th and 180th days of the experiment, 10 rats (half male and half female) were randomly selected from each group, and the blood sample was collected for routine and biochemical parameters tests of blood. The body weight of rats was measured and the vital organs including heart, liver, kidney, spleen, lung, gastrointestinal, brain, testis, and ovary organs of rats were collected. After weighing, the organ index of each organ was calculated (organ index = organ weight/body weight), and the pathological changes of liver, kidney, spleen, stomach, testis, and ovary of rats in the H group and NC group were observed.

### 2.6 Safety and efficacy evaluation of matrine on weaned piglets and its mechanism

A total of 1,200 weaned piglets were randomly divided into six groups: A, B, C, D, positive control (PC), and NC groups. The treatment of each group is shown in Table 2. Each group was divided into five columns, with 40 pigs in each column. After 7 days of adaptive feeding, the drug was administered for 34 days. During the experiment, the feed intake of piglets in each group was recorded every day. The piglets in each group were weighed on the 17 th and 34 th day of administration and the ADG, ADFI, and FCR were calculated. Twenty four hours after the last administration, 10 piglets were randomly selected from each group to collect blood and fecal samples, 8 of them were randomly selected from the NC group and the matrine group (with the best growth-promoting effect) for fecal omics analysis. Metagenomics was employed for comparative analysis in the variations in fecal flora between groups. Non-targeted metabolomics was used to analyze the differences in fecal metabolites between groups. The correlation between metagenomics, metabolomics and growth performance was analyzed by Mothur software. Blood samples were used for the tests of biochemical parameters, positive rate of PCV2 vaccine antibodies, and immunological indices. The contents of IgA, IgG, IgM, TNF-*α*, IL-1β, IL-6, IL-8, and the positive rate of PCV2 vaccine antibody in serum were detected by ELISA kit (Shanghai Enzyme-linked Biotechnology Co., Ltd.) (On the 20th day of administration, all piglets were immunized with PCV2 vaccine, therefore, in this experiment, we explored whether matrine could improve the positive rate of PCV2 vaccine antibody in piglets). Three piglets were randomly selected from each group for autopsy and the heart, liver, spleen, lung, kidney, and small intestine were collected for pathological examination.

**Table 2:** Experimental grouping and processing.

Group	Treatment
A	Basal diets+0.375 mg/kg Matrine
B	Basal diets+0.750 mg/kg Matrine
C	Basal diets+1.500 mg/kg Matrine
D	Basal diets+3.000 mg/kg Matrine
PC	Basal diets+50 mg/kg Boluohui San
NC	Basal diets

### 2.7 Blood routine and blood biochemical parameters tests

According to the experimental design, the blood of experimental animals was collected at a specific time, and a part of the blood was transferred to the EDTA blood collection tube for the conduction of the hematological parameters test. The blood routine indexes were measured by an automatic blood cell analyzer (Guilin URIT, China). Another part of the blood was transferred to a blood collection tube without anticoagulants, placed at room temperature for 15 min, centrifuged at 3500 r/min for 10 min, and serum was collected for blood biochemical parameters detection. Blood biochemical parameters were measured by an automatic biochemical analyzer (Tianjin MNCHIP, China).

### 2.8 Organ histomorphology analysis

After the duodenum, jejunum, ileum, heart, liver, spleen, lung, kidney, testis, and ovary were collected, the contents, adipose tissue and blood were removed. The collected tissues were fixed with 4% paraformaldehyde (Servicebio, China), embedded in paraffin, sectioned into 4 μm thick histological sections, and then stained with hematoxylin and eosin staining (H&E) (Solarbio, China). The examination was performed using an optical microscope (Olympus, Japan). Histopathological examination was performed by experienced pathologists.

### 2.9 Metagenome DNA extraction and shotguns sequencing

Total microbial genomic DNA samples were extracted using the OMEGA Mag-Bind Soil DNA Kit (M5635-02) (Omega Bio-Tek, Norcross, GA, United States) as per following the manufacturer’s instructions and stored at −20°C prior to further assessment. The quantity and quality of extracted DNAs were measured using a Qubit™ 4 Fluorometer, with WiFi: Q33238 (Qubit™ Assay Tubes: Q32856; Qubit™ 1X dsDNA HS Assay Kit: Q33231) (Invitrogen, United States) and agarose gel electrophoresis, respectively. The extracted microbial DNA was processed to construct metagenome shotgun sequencing libraries with insert sizes of 400 bp by using the Illumina TruSeq Nano DNA LT Library Preparation Kit. Each library was sequenced by the Illumina NovaSeq platform (Illumina, United States) with PE150 strategy at Personal Biotechnology Co., Ltd. (Shanghai, China).

### 2.10 Preparation of fecal non-target metabolomics samples

After 24 h of the last dose administration, 100 mg of samples were precisely measured and placed in a 2 mL centrifuge tube, 600 μL of MeOH [Containing 2-Amino-3-(2-chloro-phenyl)-propionic] acid (4 ppm) was added, and the vortex oscillated for 30 s. The steel balls were added into the tissue grinder and ground at 50 Hz for 120 s. After ultrasonication at room temperature for 10 min and centrifugation at 12,000 rpm at 4°C for 10 min, the supernatant was filtered through a 0.22 μm pore-sized filter, and the filtrate was added to the detection bottle for LC–MS detection.

### 2.11 Liquid chromatography conditions

The LC analysis was performed on a Vanquish UHPLC System (Thermo Fisher Scientific, United States). Chromatography was carried out with an ACQUITY UPLC^®^ HSS T3 (2.1 × 100 mm, 1.8 μm) (Waters, Milford, MA, United States). The column was maintained at 40°C. The flow rate and injection volume were set at 0.3 mL/min and 2 μL, respectively. For LC-ESI (+)-MS analysis, the mobile phases consisted of (B1) 0.1% formic acid in acetonitrile (v/v) and (A1) 0.1% formic acid in water (v/v). Separation was conducted under the following gradient: 0 ~ 1 min, 8% B1; 1 ~ 8 min, 8% ~ 98% B1; 8 ~ 10 min, 98% B1; 10 ~ 10.1 min, 98% ~ 8% B1; 10.1 ~ 12 min, 8% B1. For LC-ESI (−)-MS analysis, the analytes were carried out with (B2) acetonitrile and (A2) ammonium formate (5 mM). Separation was conducted under the following gradient: 0 ~ 1 min, 8% B2; 1 ~ 8 min, 8% ~ 98% B2; 8 ~ 10 min, 98% B2; 10 ~ 10.1 min, 98% ~ 8% B2; 10.1 ~ 12 min, 8% B2.

### 2.12 Mass spectrum conditions

Mass spectrometric detection of metabolites was performed on Orbitrap Exploris 120 (Thermo Fisher Scientific, United States) with an ESI ion source. Simultaneous MS1 and MS/MS (Full MS-ddMS2 mode, data-dependent MS/MS) acquisition was used. The parameters were as follows: sheath gas pressure, 40 arb; aux gas flow, 10 arb; spray voltage, 3.50 and −2.50 kV for ESI(+) and ESI(−), respectively; capillary temperature, 325°C; MS1 range, m/z 100–1,000; MS1 resolving power, 60,000 FWHM; number of data dependant scans per cycle, 4; MS/MS resolving power, 15,000 FWHM; normalized collision energy, 30%; dynamic exclusion time, automatic.

### 2.13 Data analysis

Excel was used to collate the data, and SPSS 26 software was used for one-way analysis of variance (one-way ANOVA) to compare the data between groups. *p* > 0.05 indicated that there was no significant difference, *p* < 0.05 indicated that the difference was significant, and different lowercase letters indicated that the difference was significant (*p* < 0.05). The data were expressed as mean ± standard deviation, and GraphPad Prism 8.0.2 (GraphPad Software, Inc., California, United States) software was used for plotting. In Spearman correlation analysis results, * indicates *p* < 0.05, and ** indicates *p* < 0.01.

## 3 Results

### 3.1 The results of acute toxicity test of matrine

The results of the acute toxicity test of matrine showed that after intragastric administration of 300 mg/kg matrine, the mice exhibited restlessness, continuous running, intense tremors and convulsion, followed by curling up, reduced activity, and squinting. All 10 mice died within 10 min and the eyeballs protruded when they died. After intragastric administration of 210 and 147 mg/kg matrine, the above poisoning symptoms could also be caused in mice, but the symptoms were alleviated with the decrease of matrine dose, and the death time was within 12 h. Although no mice died in group D, squinting and curling appeared within 1 h after administration and returned to normal condition after 3 h. There were no adverse reactions in groups E and F mice. The LD_50_ of matrine on ICR mice was calculated to be 202.54 mg/kg by JMP Clinical 17 software, and the 95% confidence interval was 176.20 ~ 233.35 mg/kg (Table 3).

**Table 3:** The results of the acute toxicity assay of matrine.

Group	Dose (mg/kg)	Number of mice	Number of dead mice	Mortality (%)	LD_50_(mg/kg)	Confidence interval (mg/kg)
A	300.00	10	10	100	202.54	176.20 ~ 233.35
B	210.00	10	4	40		
C	147.00	10	2	20		
D	102.90	10	0	0		
E	72.03	10	0	0		
F	50.42	10	0	0		

### 3.2 Blood routine and blood biochemical parameters detection of weaned rats

The results of the blood routine showed that after 90 days and 180 days of intragastric administration of matrine in SD-weaned rats, most of the blood routine indexes in each group were within the reference range, but a small number of indexes such as MON % were abnormal. Specifically, MON % was normal in group H but abnormal in NC and L groups, MCHC was abnormal in all groups at 90 days, indicating that these abnormalities were not caused by matrine, but may be caused by inaccurate reference range or other factors (The same problem exists in the following biochemical parameters detection), ultimately indicating that matrine had no effect on the blood routine of SD rats in the dose range of 24.5 ~ 50 mg/kg (Table 4). The results of blood biochemical parameters showed that after 90 days and 180 days of intragastric administration of matrine in SD-weaned rats, most of the biochemical indexes in each group were within the reference range. Although there was no accurate reference range for AST/ALT, BUN/Cr, and Ca×P, statistical analysis showed that there was no significant difference between the H group and the NC group, indicating that matrine did not affect the blood biochemical parameters of SD rats in the dose range of 24.5 ~ 50 mg/kg (Table 5).

**Table 4:** Results of the blood routine test.

Parameter	Reference range	Detection time	L	M	H	NC
WBC, 10^9^·L^−1^	1.9 ~ 16.8	90 d	5.69 ± 2.32^a^	2.27 ± 2.46^b^	6.62 ± 2.90^a^	5.46 ± 2.35^a^
		180 d	7.37 ± 4.13	5.35 ± 2.14	7.17 ± 3.49	7.06 ± 3.58
LYM%	40 ~ 88.9	90 d	58.56 ± 6.20	56.46 ± 5.02	57.50 ± 6.29	57.47 ± 4.42
		180 d	55.64 ± 13.20	61.04 ± 6.79	60.15 ± 8.35	52.94 ± 15.29
MON%	12.0 ~ 18.0	90 d	11.90 ± 1.69^b^	14.15 ± 3.46^ab^	16.43 ± 2.45^a^	11.88 ± 1.98^b^
		180 d	12.99 ± 2.29	13.00 ± 2.31	11.52 ± 3.15	13.69 ± 2.86
NEU%	7.3 ~ 50	90 d	25.39 ± 6.43	26.01 ± 6.80	22.72 ± 5.46	27.13 ± 4.80
		180 d	28.21 ± 14.63	22.53 ± 6.09	24.91 ± 10.00	28.92 ± 16.56
EOS%	0 ~ 7	90 d	3.82 ± 1.06	3.10 ± 1.12	3.07 ± 0.48	3.19 ± 1.8830
		180 d	3.05 ± 1.07	3.2200 ± 0.57	3.1744 ± 0.76	4.0230 ± 1.44
BASO%	0 ~ 1.5	90 d	0.33 ± 0.19	0.28 ± 0.17	0.29 ± 0.23	0.33 ± 0.21
		180 d	0.11 ± 0.07^b^	0.21 ± 0.14^ab^	0.26 ± 0.22^ab^	0.43 ± 0.33^a^
LYM, 10^9^·L^−1^	0.91 ~ 12.2	90 d	3.35 ± 1.39^ab^	1.37 ± 1.56^b^	3.91 ± 2.08^a^	3.11 ± 1.34^ab^
		180 d	3.63 ± 0.76	3.27 ± 1.38	4.36 ± 2.24	3.25 ± 0.49
MON, 10^9^·L^−1^	0.08 ~ 2.3	90 d	0.67 ± 0.27^ab^	0.32 ± 0.36^b^	1.10 ± 0.55^a^	0.66 ± 0.31^b^
		180 d	0.95 ± 0.53	0.69 ± 0.30	0.86 ± 0.47	0.97 ± 0.57
NEU, 10^9^·L^−1^	0.5 ~ 6.3	90 d	1.44 ± 0.73^a^	0.51 ± 0.50^b^	1.40 ± 0.32^a^	1.48 ± 0.66^a^
		180 d	2.59 ± 3.27	1.21 ± 0.59	1.70 ± 0.94	2.57 ± 3.11
EOS, 10^9^·L^−1^	0 ~ 1.01	90 d	0.22 ± 0.11^a^	0.06 ± 0.06^b^	0.20 ± 0.09^a^	0.19 ± 0.15^a^
		180 d	0.19 ± 0.04	0.17 ± 0.07	0.23 ± 0.11	0.25 ± 0.06
BASO, 10^9^·L^−1^	0 ~ 0.2	90 d	0.02 ± 0.01	0.01 ± 0.01	0.02 ± 0.01	0.02 ± 0.01
		180 d	0.01 ± 0.01^b^	0.01 ± 0.01^ab^	0.02 ± 0.01^ab^	0.03 ± 0.02^a^
ALY%	0 ~ 99.99	90 d	0.94 ± 0.19^ab^	0.78 ± 0.17^ab^	1.04 ± 0.37^a^	0.72 ± 0.27^b^
		180 d	1.06 ± 0.42^ab^	1.18 ± 0.43^ab^	1.31 ± 0.35^a^	0.92 ± 0.31^b^
ALY, 10^9^·L^−1^	0 ~ 99.999	90 d	0.05 ± 0.02^ab^	0.02 ± 0.02^b^	0.07 ± 0.06^a^	0.04 ± 0.02^ab^
		180 d	0.07 ± 0.02^ab^	0.06 ± 0.04^ab^	00.09 ± 0.05^a^	0.06 ± 0.01^b^
LIC%	0 ~ 99.99	90 d	0.16 ± 0.18	0.19 ± 0.22	0.25 ± 0.40	0.63 ± 0.69
		180 d	0.29 ± 0.25	0.16 ± 0.11	0.29 ± 0.35	0.43 ± 0.65
LIC#, 10^9^·L^−1^	0 ~ 99.999	90 d	0.01 ± 0.01	0.01 ± 0.01	0.01 ± 0.02	0.04 ± 0.05
		180 d	0.03 ± 0.04	0.01 ± 0.01	0.02 ± 0.02	0.05 ± 0.10
RBC, 10^12^·L^−1^	5 ~ 9.8	90 d	8.99 ± 0.61^a^	7.87 ± 0.65^b^	8.33 ± 1.10^ab^	7.38 ± 0.67^b^
		180 d	7.32 ± 0.47^b^	7.58 ± 1.09^ab^	8.20 ± 0.87^a^	7.34 ± 0.42^b^
HGB, g·L^−1^	110 ~ 170	90 d	203.67 ± 13.26^a^	174.83 ± 11.86^b^	179.75 ± 14.88^b^	164.29 ± 13.56^b^
		180 d	155.22 ± 13.07^ab^	156.50 ± 11.76^ab^	163.22 ± 12.33^a^	152.30 ± 9.18^b^
HCT/%	32 ~ 53	90 d	53.20 ± 3.90^a^	45.70 ± 3.82^b^	46.73 ± 5.09^b^	42.00 ± 3.74^b^
		180 d	43.10 ± 3.64^b^	44.32 ± 5.27^ab^	47.51 ± 4.04^a^	41.95 ± 2.74^b^
MCV/fL	50 ~ 65	90 d	59.20 ± 0.99^a^	58.13 ± 0.75^ab^	56.34 ± 1.80^b^	57.03 ± 1.53^b^
		180 d	58.88 ± 2.20	58.77 ± 2.59	58.16 ± 1.85	57.23 ± 2.38
MCH/pg	16 ~ 23	90 d	22.57 ± 0.45	22.18 ± 0.48	21.65 ± 1.37	22.21 ± 1.39
		180 d	21.12 ± 1.12^a^	20.75 ± 1.67^ab^	19.93 ± 0.84^b^	20.68 ± 0.89^ab^
MCHC, g·L^−1^	310 ~ 370	90 d	382.33 ± 4.41	382.50 ± 8.02	384.88 ± 13.59	390.43 ± 16.60
		180 d	359.11 ± 9.01^a^	354.00 ± 19.65^ab^	343.22 ± 5.80^b^	362.50 ± 6.06^a^
RDW-CV/%	11 ~ 16	90 d	12.40 ± 0.50	12.93 ± 1.11	12.50 ± 0.88	12.61 ± 0.81
		180 d	13.31 ± 0.70	13.45 ± 0.67	13.10 ± 0.74	13.50 ± 1.08
PLT, g·L^−1^	250 ~ 1,500	90 d	742.17 ± 132.13	766.33 ± 221.42	768.75 ± 159.61	639.00 ± 234.85
		180 d	1014.44 ± 136.13^a^	901.50 ± 285.80^a^	610.78 ± 335.82^b^	1036.70 ± 283.71^a^
MPV/fL	4.8 ~ 7	90 d	5.57 ± 0.10	5.67 ± 0.37	5.54 ± 0.24	5.79 ± 0.31
		180 d	6.00 ± 0.22	6.28 ± 0.56	6.09 ± 0.31	5.96 ± 0.33

The reference range of the parameters in the table is provided by Guilin URIT, China. Different letters indicate significant differences between groups, *p* < 0.05.

**Table 5:** The results of blood biochemical parameters tests.

Parameter	Reference range	Detection time	L	M	H	NC
TP, g·L^−1^	53 ~ 82	90 d	71.12 ± 4.26	74.03 ± 8.18	69.55 ± 6.08	71.29 ± 13.53
		180 d	69.72 ± 2.80^d^	92.63 ± 4.10^a^	77.35 ± 2.26^c^	83.98 ± 5.78^b^
ALB, g·L^−1^	30 ~ 48	90 d	45.58 ± 3.55	47.13 ± 5.34	44.06 ± 4.61	45.96 ± 8.95
		180 d	38.74 ± 0.94^c^	50.90 ± 1.01^a^	48.10 ± 2.63^ab^	44.82 ± 3.81^b^
GLB, g·L^−1^	10 ~ 50	90 d	25.53 ± 1.56	26.90 ± 3.34	25.49 ± 2.66	25.33 ± 4.68
		180 d	30.98 ± 3.69^b^	41.73 ± 3.37^a^	29.25 ± 3.23^b^	39.16 ± 3.08^a^
TBIL, umol·L^−1^	0 ~ 12	90 d	3.86 ± 1.35	3.10 ± 0.99	3.44 ± 1.15	3.41 ± 0.93
		180 d	2.59 ± 0.52	3.29 ± 1.03	3.29 ± 0.70	3.71 ± 1.94
ALT, U·L^−1^	20 ~ 61	90 d	53.33 ± 12.01	56.83 ± 14.22	47.50 ± 6.39	55.57 ± 33.58
		180 d	72.60 ± 31.44	86.33 ± 9.29	90.50 ± 29.08	82.60 ± 21.95
AST, U·L^−1^	39 ~ 164	90 d	145.00 ± 30.26	118.50 ± 26.99	126.00 ± 18.98	148.43 ± 86.17
		180 d	143.40 ± 58.27	197.67 ± 17.01	193.00 ± 64.22	206.40 ± 73.17
AST/ALT		90 d	2.76 ± 0.54	2.12 ± 0.35	2.68 ± 0.45	2.79 ± 0.98
		180 d	1.94 ± 0.32	2.29 ± 0.09	2.17 ± 0.52	2.57 ± 0.83
ALP, U·L^−1^	16 ~ 302	90 d	68.00 ± 26.50	64.17 ± 32.57	59.88 ± 23.43	60.86 ± 22.76
		180 d	126.40 ± 33.31^b^	189.67 ± 8.33^a^	132.00 ± 27.31^b^	132.40 ± 14.29^b^
TBA, μmol·L^−1^	0 ~ 15	90 d	13.82 ± 3.92	10.78 ± 3.65	12.65 ± 3.99	12.93 ± 8.10
		180 d	42.46 ± 33.28	56.03 ± 6.11	51.00 ± 33.52	31.94 ± 3.45
AMS, U·L^−1^	200 ~ 2,500	90 d	675.00 ± 157.31	716.17 ± 141.32	733.88 ± 223.19	715.57 ± 156.22
		180 d	688.80 ± 98.60^b^	1061.67 ± 147.87^a^	1085.50 ± 241.40^a^	957.20 ± 214.06^a^
TG, mmol·L^−1^	0.29 ~ 1.22	90 d	0.94 ± 0.19	0.95 ± 0.16	1.03 ± 0.25	0.98 ± 0.14
		180 d	1.06 ± 0.37^b^	1.90 ± 0.39^a^	1.36 ± 0.12^ab^	1.40 ± 0.70^ab^
Chol, mmol·L^−1^	0.52 ~ 2.38	90 d	3.22 ± 0.74^ab^	3.05 ± 0.83^ab^	3.73 ± 0.93^a^	2.57 ± 0.60^b^
		180 d	3.27 ± 0.56^b^	5.33 ± 0.19^a^	4.70 ± 0.74^a^	3.61 ± 0.77^b^
GLU, mmol·L^−1^	2.78 ~ 7.92	90 d	4.61 ± 0.75	5.62 ± 1.17	5.65 ± 2.46	5.36 ± 1.38
		180 d	5.00 ± 0.38^b^	5.57 ± 0.21^ab^	5.79 ± 1.10^ab^	6.35 ± 0.99^a^
Cr, umol·L^−1^	17 ~ 70	90 d	41.33 ± 9.77	42.17 ± 5.42	35.50 ± 5.15	45.29 ± 21.05
		180 d	51.80 ± 22.93	37.67 ± 7.64	44.25 ± 21.44	39.60 ± 9.94
BUN, mmol·L^−1^	1.7 ~ 10.2	90 d	4.48 ± 0.91^b^	4.22 ± 0.48^b^	5.01 ± 0.39^ab^	5.79 ± 1.24^a^
		180 d	8.61 ± 1.31	8.61 ± 1.84	7.14 ± 1.61	8.61 ± 1.24
BUN/Cr		90 d	27.17 ± 4.40^b^	25.00 ± 2.10^b^	35.88 ± 6.60^a^	36.00 ± 11.24^a^
		180 d	35.20 ± 14.69	57.00 ± 10.44	47.75 ± 24.12	52.40 ± 15.57
T-CO2, mmol·L^−1^	12.5 ~ 32	90 d	19.67 ± 1.75	20.83 ± 4.62	19.38 ± 4.03	18.00 ± 1.73
		180 d	30.20 ± 1.92^a^	21.33 ± 3.79^b^	18.75 ± 2.36^b^	31.60 ± 4.22^a^
Ca, mmol·L^−1^	1.81 ~ 3.54	90 d	2.55 ± 0.15^ab^	2.72 ± 0.26^a^	2.54 ± 0.17^ab^	2.43 ± 0.35^b^
		180 d	2.26 ± 0.12^c^	2.81 ± 0.05^a^	2.59 ± 0.12^b^	2.69 ± 0.15^ab^
P, mmol·L^−1^	1.07 ~ 3.55	90 d	2.61 ± 0.25	2.78 ± 0.72	2.86 ± 0.56	2.42 ± 0.34
		180 d	2.43 ± 0.25^b^	3.26 ± 0.32^a^	3.31 ± 0.32^a^	3.04 ± 0.11^a^
Ca×P, mg·dL^−1^		90 d	82.33 ± 7.06	93.83 ± 25.20	90.00 ± 17.09	74.00 ± 19.36
		180 d	68.60 ± 10.55^b^	113.33 ± 10.21^a^	106.00 ± 12.52^a^	101.60 ± 9.24^a^
Mg, mmol·L^−1^	0.66 ~ 1.81	90 d	1.15 ± 0.12	1.12 ± 0.27	1.13 ± 0.09	1.10 ± 0.17
		180 d	1.10 ± 0.05^c^	1.60 ± 0^a^	1.45 ± 0.15^ab^	1.34 ± 0.10^b^

The reference range of the parameters in the table is provided by Tianjin MNCHIP, China, and the blank indicates that there is no accurate reference range. Different letters indicate significant differences between groups, *p* < 0.05.

### 3.3 Organ index detection of weaned rats

After 90 days of administration of matrine in SD-weaned rats, compared with the NC group the lung organ index of male rats in the L group, the gastrointestinal organ index of female rats in the L group, and the gastrointestinal organ index of male rats in the H group were significantly increased (*p* < 0.05). There was no difference in other organ indexes between groups. After 180 days of administration of matrine to SD-weaned rats, compared with the NC group, the lung organ index of male rats in the L group was significantly increased (*p* < 0.05), and there was no difference between the other organ indexes (Table 6).

**Table 6:** The results of the organ index test in rats.

Organ	Time	NC—♂	L—♂	M—♂	H—♂	NC—♀	L—♀	M—♀	H—♀
Heart	90d	0.31 ± 0.03	0.30 ± 0.03	0.34 ± 0.05	0.32 ± 0.03	0.35 ± 0.02	0.34 ± 0.01	0.35 ± 0.01	0.35 ± 0.05
	180d	0.31 ± 0.03	0.33 ± 0.0	0.33 ± 0.04	0.29 ± 0.02	0.39 ± 0.06	0.40 ± 0.05	0.35 ± 0.01	0.41 ± 0.04
Liver	90d	2.65 ± 0.23	2.55 ± 0.21	2.59 ± 0.26	2.81 ± 0.23	2.79 ± 0.25	2.77 ± 0.15	2.58 ± 0.04	2.74 ± 0.21
	180d	3.08 ± 0.23	3.04 ± 0.17	3.32 ± 0.35	3.21 ± 0.43	3.42 ± 0.32	3.47 ± 0.14	3.64 ± 0.20	3.19 ± 0.28
Spleen	90d	0.18 ± 0.01	0.17 ± 0.01	0.18 ± 0.02	0.18 ± 0.03	0.22 ± 0.03	0.23 ± 0.01	0.21 ± 0.04	0.21 ± 0.04
	180d	0.15 ± 0.02	0.15 ± 0.02	0.16 ± 0.2	0.14 ± 0.02	0.21 ± 0.05	0.21 ± 0.02	0.21 ± 0.01	0.24 ± 0.04
Lung	90d	0.38 ± 0.07^b^	0.56 ± 0.17^a^	0.44 ± 0.07^ab^	0.49 ± 0.18^ab^	0.61 ± 0.13	0.62 ± 0.09	0.73 ± 0.07	0.59 ± 0.08
	180d	0.39 ± 0.04^b^	0.44 ± 0.04^a^	0.42 ± 0.04^ab^	0.41 ± 0.02^ab^	0.57 ± 0.05	0.55 ± 0.08	0.66 ± 0.19	0.56 ± 0.11
Kidney	90d	0.64 ± 0.14	0.66 ± 0.04	0.71 ± 0.10	0.66 ± 0.07	0.67 ± 0.14	0.62 ± 0.01	0.70 ± 0.00	0.67 ± 0.03
	180d	0.70 ± 0.02	0.68 ± 0.08	0.73 ± 0.08	0.69 ± 0.03	0.82 ± 0.15	0.79 ± 0.08	0.83 ± 0.13	0.77 ± 0.08
Gastrointestinal	90d	3.54 ± 0.20^b^	4.04 ± 0.26^ab^	3.90 ± 0.55^ab^	4.15 ± 0.36^a^	4.62 ± 0.45^b^	6.37 ± 1.55^a^	4.90 ± 0.76^ab^	5.92 ± 0.64^ab^
	180d	6.39 ± 0.50	6.15 ± 0.19	5.96 ± 1.22	5.92 ± 0.13	7.72 ± 0.59	7.78 ± 1.64	8.25 ± 3.15	7.42 ± 0.35
Testis/ovary	90d	0.77 ± 0.03	0.85 ± 0.14	0.86 ± 0.03	0.80 ± 0.04	0.12 ± 0.14	0.06 ± 0.01	0.08 ± 0.01	0.09 ± 0.03
	180d	0.76 ± 0.08^ab^	0.83 ± 0.10^a^	0.77 ± 0.05^ab^	0.70 ± 0.05^b^	0.06 ± 0.01	0.07 ± 0.01	0.06 ± 0.01	0.09 ± 0.07
Brain	90d	0.37 ± 0.04	0.38 ± 0.04	0.43 ± 0.05	0.41 ± 0.04	0.72 ± 0.06	0.67 ± 0.05	0.63 ± 0.04	0.64 ± 0.01
	180d	0.31 ± 0.05	0.36 ± 0.04	0.39 ± 0.03	0.34 ± 0.03	0.61 ± 0.11	0.56 ± 0.09	0.43 ± 0.07	0.53 ± 0.06

Different letters indicate significant differences between groups, *P* < 0.05.

### 3.4 Morphological characteristics of some organs of weaned rats

**Figure 1:**
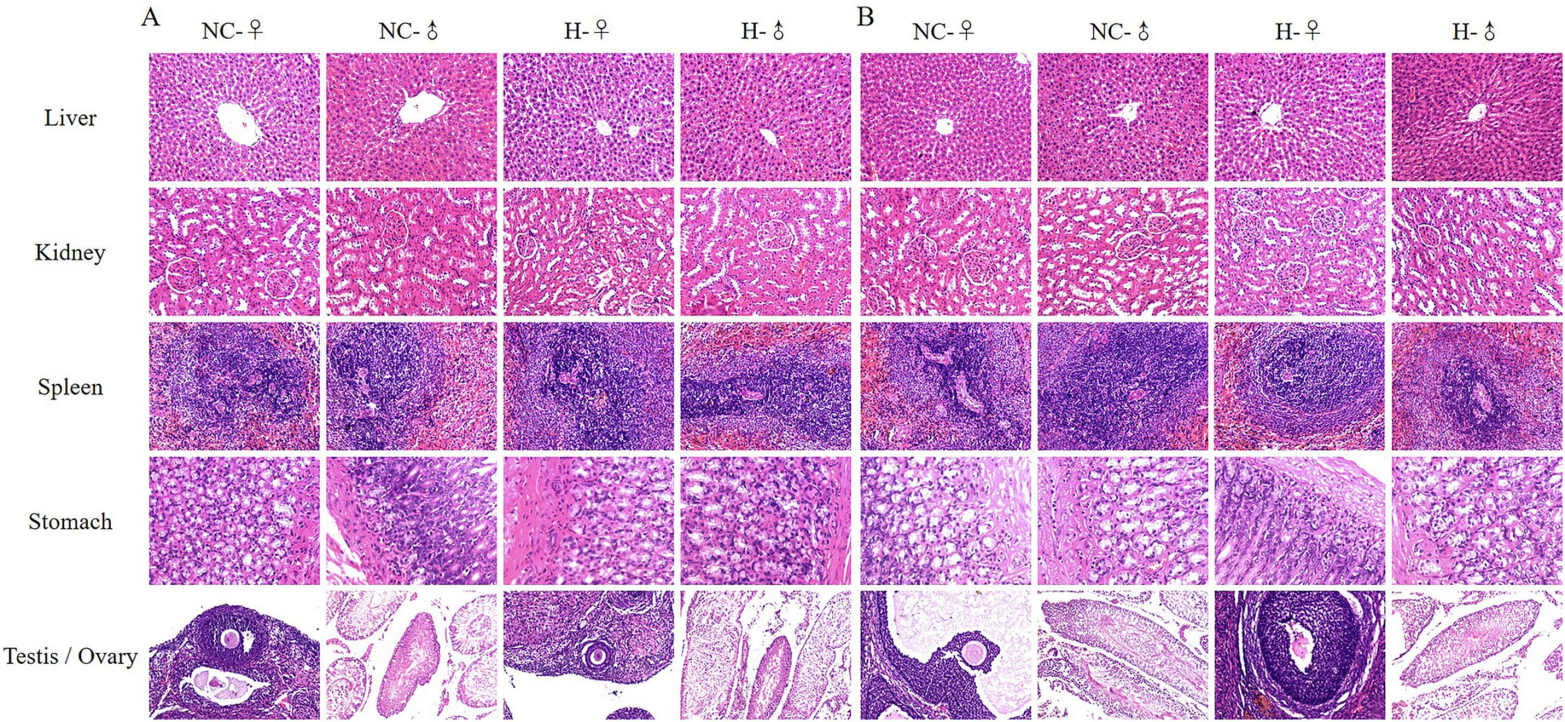
Representative images of some rat organs by H&E staining. **(A)** H&E staining of organs after 90 days of matrine gavage. **(B)** H&E staining of organs after 180 days of matrine gavage.

The results of organ morphology in rats showed that after intragastric administration of matrine in SD-weaned rats for 90 days (Figure 1A) and 180 days (Figure 1B), compared with the NC group, there was no obvious abnormality in the liver, kidney, spleen, stomach and reproductive organs (testis and ovary) of the H group. The specific performance was as follows: the liver cells were arranged in a regular cord-like shape, and a clear central vein can be seen. The liver cell cords were arranged radially around the central vein. The liver cytoplasm was uniformly purple-red, and the liver nucleus was large and round. The morphological structure of kidney tissue was normal and clear, the arrangement of renal tubules and glomeruli was regular and the space, volume, and glomerular volume of the renal capsule were normal. The morphological structure of the spleen tissue was normal, boundary of the white pulp area was obvious and clear, and lymphocytes of the lymphatic sheath around the central artery were closely arranged. The morphological structure of gastric mucosa was normal, the glands were immaculately organized, and the cell density of lamina propria was normal. The morphology of the testis was complete and clear, structure was normal, the morphology of the seminiferous tubules was normal, and there were spermatogonia, primary spermatocytes, and supporting cells in the lumen. The cells were arranged in an orderly manner without obvious lesions. The follicles in the ovary developed completely, the morphology of follicles at all levels was normal, the level of granulosa cells was rich, and there was no obvious lesion. The above results showed that after intragastric administration of 50 mg/kg matrine to SD-weaned rats for 90 days and 180 days, there were no abnormalities in the liver, kidney, spleen, stomach, and reproductive organs of female and male rats, indicating that 24.5 ~ 50 mg/kg matrine can be safely applied to SD rats.

### 3.5 Matrine improved the growth performance of weaned piglets

The results of body weight and feed intake of weaned piglets showed that during 1–17 days the FCR of the NC group and C group were 1.44 and 1.37, respectively. Compared with the NC group, the C group decreased by 4.86%, and other groups did not promote the growth of piglets. During 18 ~ 34 days, the FCR of NC, A, B, C, D, and PC groups were 1.38, 1.33, 1.32, 1.33, 1.40, and 1.37, respectively. Compared with the NC group, the FCR of A, B, C, and PC groups decreased by 3.62, 4.35, 3.62, and 0.72%, respectively, while that of the D group increased by 1.45%. During 1–34 days, the FCR of the NC, A, B, C, D, and PC groups were 1.40, 1.40, 1.38, 1.35, 1.44, and 1.39, respectively. Compared with the NC group, the A, B, C, and PC groups decreased by 0, 1.43, 3.57, and 0.71%, respectively, and the D group increased by 2.86% (Table 7). In order to improve the feed utilization rate and growth efficiency of piglets, the farm administrator normally changes the feed of piglets from powder feed to granular feed during the feeding stage of piglets. The farm where this experiment was located also changed the feed on the 20th day of the experiment. The replacement of feed will lead to clinical symptoms such as diarrhea in piglets. During the breeding process, it was found that the diarrhea symptoms caused by the replacement of feed in piglets. It is worth noting that compared with group B, the diarrhea symptoms in group C and group D were more serious. In summary, the addition of 0.75 mg/kg matrine to the feed had the best growth-promoting effect.

**Table 7:** Effects of matrine on growth performance of weaned piglets.

Time	Parameter	A	B	C	D	PC	NC
D 1	AW/kg	9.31 ± 1.07	9.34 ± 0.94	9.32 ± 0.87	9.21 ± 1.72	9.22 ± 1.71	9.21 ± 1.62
D 17	AW/kg	15.74 ± 1.08	15.81 ± 0.88	16.19 ± 1.15	15.41 ± 2.00	15.83 ± 2.21	15.76 ± 1.82
D 34	AW/kg	24.71 ± 2.05	25.03 ± 2.09	25.07 ± 1.54	23.73 ± 3.30	24.35 ± 3.62	24.13 ± 3.10
D 1 ~ D 17	ADG/kg	0.38 ± 0.02	0.38 ± 0.01	0.40 ± 0.02	0.36 ± 0.02	0.39 ± 0.04	0.39 ± 0.02
	ADFI/kg	0.56 ± 0.03	0.56 ± 0.03	0.56 ± 0.03	0.53 ± 0.04	0.56 ± 0.04	0.55 ± 0.03
	FCR	1.49 ± 0.11	1.47 ± 0.09	1.37 ± 0.03	1.51 ± 0.08	1.44 ± 0.08	1.44 ± 0.08
D 18 ~ D 34	ADG/kg	0.53 ± 0.06	0.54 ± 0.07	0.52 ± 0.03	0.49 ± 0.08	0.50 ± 0.10	0.49 ± 0.08
	ADFI/kg	0.70 ± 0.04	0.71 ± 0.03	0.69 ± 0.03	0.68 ± 0.08	0.67 ± 0.06	0.67 ± 0.06
	FCR	1.33 ± 0.10	1.32 ± 0.14	1.33 ± 0.05	1.40 ± 0.08	1.37 ± 0.19	1.38 ± 0.15
D 1 ~ D 34	ADG/kg	0.45 ± 0.03	0.46 ± 0.04	0.46 ± 0.03	0.43 ± 0.05	0.45 ± 0.06	0.44 ± 0.05
	ADFI/kg	0.63 ± 0.03	0.64 ± 0.03	0.63 ± 0.03	0.62 ± 0.06	0.61 ± 0.05	0.61 ± 0.04
	FCR	1.40 ± 0.04	1.38 ± 0.05	1.35 ± 0.04	1.44 ± 0.06	1.39 ± 0.12	1.40 ± 0.07

### 3.6 Matrine had no effect on the immune indexes of weaned piglets

**Figure 2:**
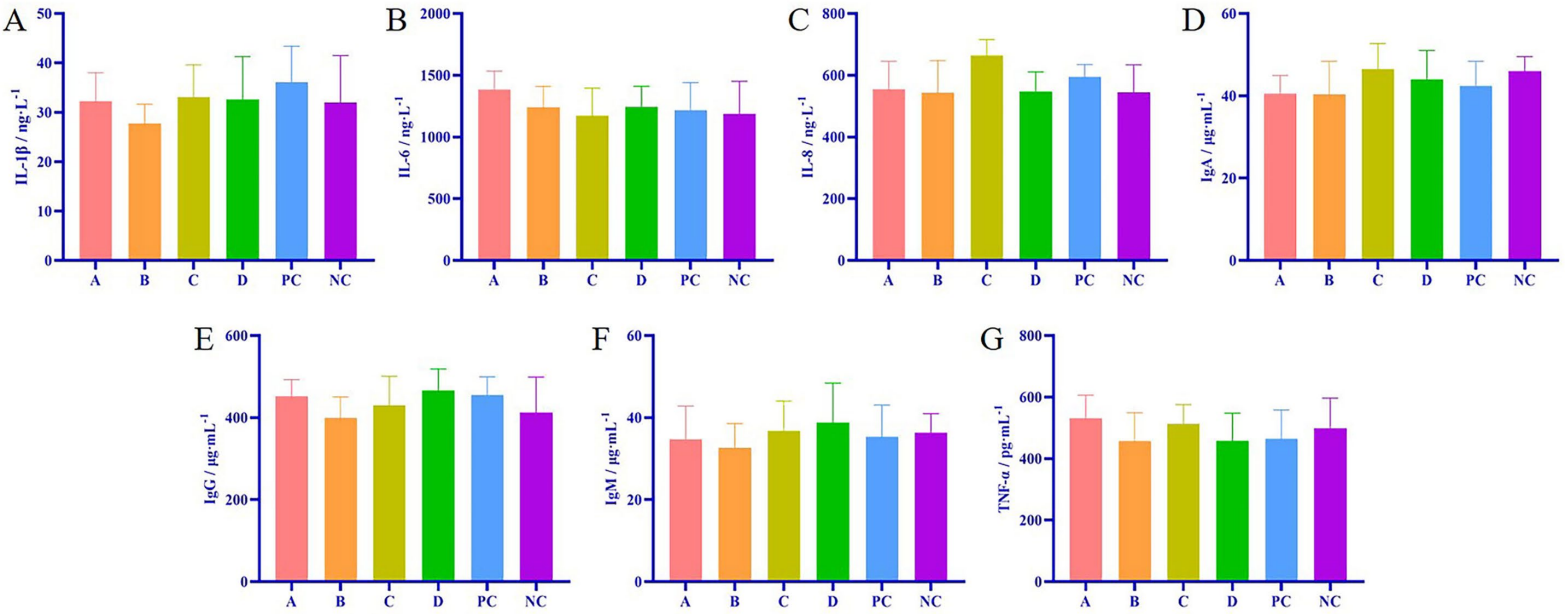
Effects of matrine on immune indexes of weaned piglets. **(A)** The detection of IL-1β in serum of weaned piglets. **(B)** The detection of IL-6 in serum of weaned piglets. **(C)** The detection of IL-8 in serum of weaned piglets. **(D)** The detection of IgA in serum of weaned piglets. **(E)** The detection of IgG in serum of weaned piglets. **(F)** The detection of IgM in serum of weaned piglets. **(G)** The detection of TNF-*α* in serum of weaned piglets.

The results of ELISA showed that there was no significant difference in the contents of IL-1β, IL-6, IL-8, IgA, IgG, IgM, and TNF-*α* in the serum of each matrine group and the positive control group compared with the NC group (*p* > 0.05) (Figure 2), indicating that matrine did not affect the immune indexes of weaned piglets.

### 3.7 Blood biochemical parameters detection in weaned piglets

The results of blood biochemical indexes showed that most of the biochemical parameters of each group were within the reference range after 34 days of adding matrine to the feed of weaned piglets. However, the AMS, ALP, and CK of group D were within the reference range, but in other low-dose groups were not within the reference range, indicating that the abnormality was not caused by matrine. In addition, the GLB, P, TP, and GLU of the NC group were not within the reference range, and there was no significant difference in GLB, TP, and GLU between the NC group and the matrine group (*p* > 0.05), indicating that the anomaly may be caused by inaccurate reference range and other factors. In summary, adding 0.375 ~ 3 mg/kg matrine to the feed for 34 consecutive days will not have a greater impact on the biochemical indicators of weaned piglets (Table 8).

**Table 8:** Blood biochemical test results of weaned piglets.

Parameter	Reference range	A	B	C	D	PC	NC
ALB, g·L^−1^	18 ~ 38	21.89 ± 8.73^ab^	26.56 ± 5.34^a^	24.66 ± 4.89^ab^	19.02 ± 10.87^ab^	20.33 ± 6.06^ab^	18.10 ± 6.57^b^
TP, g·L^−1^	46 ~ 62	38.38 ± 15.49^ab^	45.24 ± 11.96^a^	44.59 ± 10.27^a^	33.16 ± 18.39^ab^	35.86 ± 9.49^ab^	31.54 ± 10.06^b^
GLB, g·L^−1^	15 ~ 35	16.49 ± 7.24^ab^	18.68 ± 8.79^ab^	19.93 ± 6.55^a^	14.14 ± 9.05^ab^	15.52 ± 4.04^ab^	13.44 ± 4.41^b^
Ca, mmol·L^−1^	1.3 ~ 3	1.75 ± 0.54^b^	2.15 ± 0.36^a^	2.00 ± 0.21^a^	1.80 ± 0.36^ab^	1.85 ± 0.20^ab^	1.59 ± 0.37^b^
GLU, mmol·L^−1^	3.3 ~ 8.4	2.24 ± 1.13	3.20 ± 0.91	2.76 ± 1.48	2.01 ± 1.49	2.53 ± 1.58	2.27 ± 1.40
BUN, mmol·L^−1^	2.1 ~ 10.7	2.76 ± 1.12^b^	3.20 ± 0.73^b^	5.43 ± 3.90^a^	3.29 ± 0.86^b^	2.82 ± 0.58^b^	2.73 ± 0.66^b^
P, mmol·L^−1^	1.16 ~ 3.55	5.23 ± 1.39^bc^	6.35 ± 0.94^a^	6.28 ± 1.04^a^	5.91 ± 1.71^abc^	6.07 ± 0.68^ab^	4.91 ± 1.05^c^
AMS, U·L^−1^	500 ~ 1,600	1471.89 ± 898.13^ab^	1758.25 ± 400.76^a^	1659.40 ± 794.66^a^	916.00 ± 766.51^bc^	1421.33 ± 547.44^ab^	753.50 ± 232.95^c^
Chol, mmol·L^−1^	1.3 ~ 3.6	2.74 ± 1.24^b^	3.14 ± 0.52^ab^	3.92 ± 1.23^a^	3.00 ± 1.06^ab^	2.53 ± 0.63^b^	2.35 ± 1.05^b^
ALT, U·L^−1^	0 ~ 66	57.67 ± 29.74	62.50 ± 24.99	57.50 ± 20.94	40.00 ± 21.28	50.78 ± 13.03	45.88 ± 16.15
TBIL, μmol·L^−1^	0 ~ 17.1	5.91 ± 3.05	6.73 ± 2.32	8.68 ± 5.04	7.84 ± 4.84	4.90 ± 4.84	6.41 ± 4.87
ALP, U·L^−1^	41 ~ 176.1	142.33 ± 70.69^ab^	189.63 ± 76.07^a^	186.20 ± 48.96^a^	103.20 ± 71.78^b^	158.11 ± 85.19^ab^	119.75 ± 76.70^ab^
Cr, μmol·L^−1^	50 ~ 195	76.33 ± 25.05	86.38 ± 20.45	84.60 ± 15.31	83.60 ± 21.10	84.78 ± 7.68	77.00 ± 11.45
BUN/Cr		9.11 ± 2.09^b^	9.25 ± 1.58^b^	13.00 ± 13.00^a^	10.40 ± 3.85^ab^	8.22 ± 1.86^b^	9.00 ± 1.93^b^
CK, U/L	50 ~ 689.4	624.56 ± 559.99^b^	848.00 ± 285.56^b^	840.80 ± 697.94^b^	1616.40 ± 1201.62^a^	682.00 ± 331.52^b^	447.63 ± 66.56^b^

The reference range of the parameters in the table is provided by Tianjin MNCHIP, China, and the blank indicates that there is no accurate reference range. Different letters indicate significant differences between groups, *P* < 0.05.

### 3.8 Matrine increased the positive rate of PCV2 vaccine antibody in weaned piglets

The results of the PCV2 antibody in the serum of piglets detected by ELISA showed that compared with the NC group, the positive rates of PCV2 vaccine antibody in groups A and B increased by 27 and 33.9%, respectively. Compared with the PC group, the positive rates of PCV2 antibody in groups A and B increased by 18.1 and 25%, respectively. The results demonstrated that 0.375 mg/kg and 0.75 mg/kg matrine could increase the positive rate of the PCV2 vaccine antibody and the effect was better than that of Boluohui San (Table 9).

**Table 9:** Effect of matrine on the positive rate of PCV2 vaccine antibody in weaned piglets.

Antibody status of PCV2 vaccine	A	B	C	D	PC	NC
Positive	55.6%	62.5%	11.1%	20.0%	37.5%	28.6%
Negative	11.1%	12.5%	55.6%	60.0%	50.0%	71.4%
Indeterminate	33.3%	25.0%	33.3%	20.0%	12.5%	0%

### 3.9 Morphological characteristics of some organs of weaned piglets

**Figure 3:**
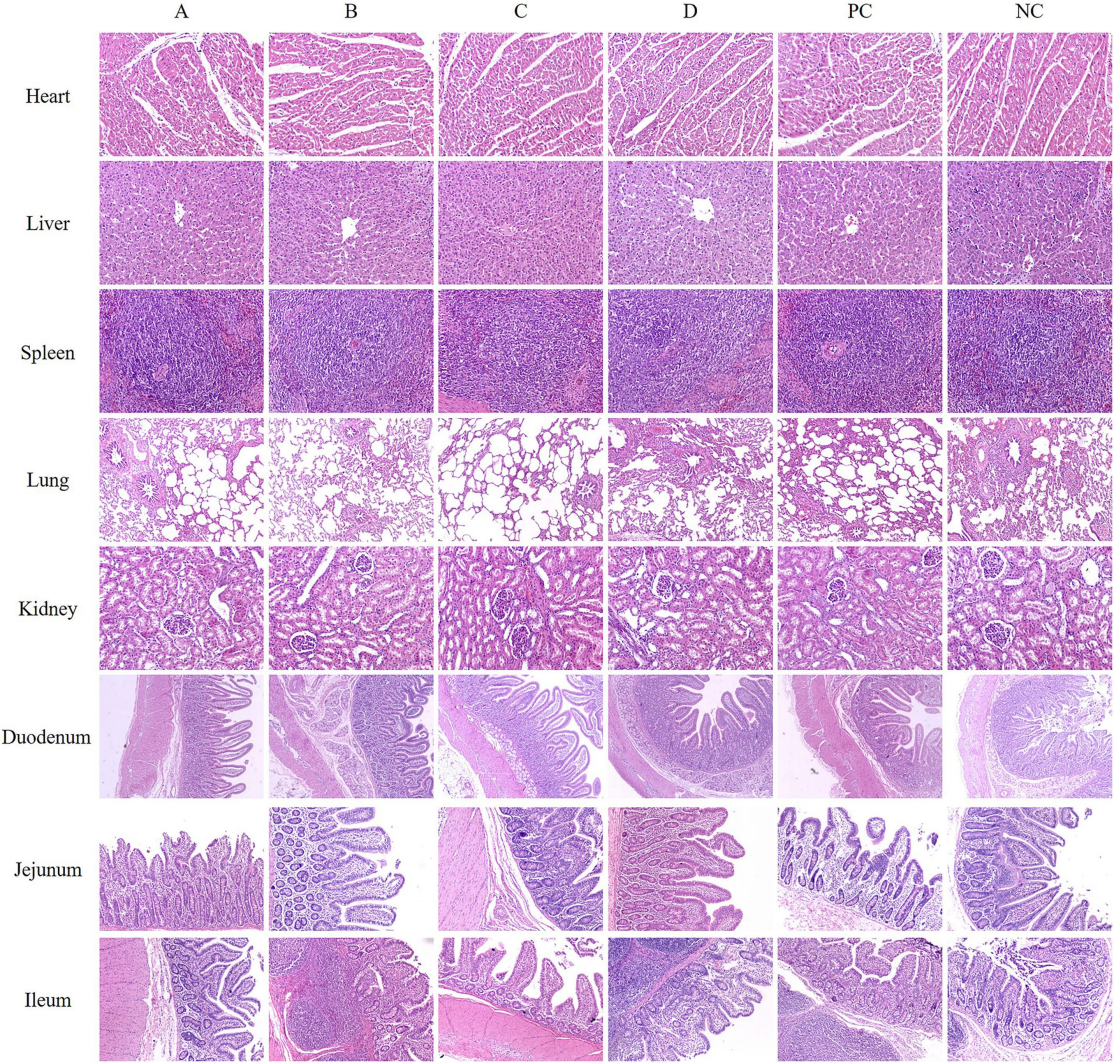
Representative images of some piglet organs by H&E staining. (A) Basal diets+0.375 mg/kg MT. (B) Basal diets+0.750 mg/kg MT. (C) Basal diets+1.500 mg/kg MT. (D) Basal diets+3.000 mg/kg MT. (PC) Basal diets+50 mg/kg BS. (NC) Basal diets.

The results of hematoxylin and eosin staining (H&E) of some organs of piglets in each group showed that compared with the NC group, there was no abnormality in the morphology of the heart, liver, spleen, kidney, and small intestine in all matrine groups and PC group. The precised manifestations are as follows: A normal cardiac tissue structure was characterized by the arrangement of myocardial cells in parallel, clear structure, regular morphology, uniformly colored cytoplasm, and a distinct nucleus, and there was no inflammatory cell infiltration and other pathological changes; The hepatic tissue exhibited normal morphology, with liver cells regularly distributed, a consistently crimson cytoplasm, a large and spherical liver cell nucleus, and a clearly visible central vein. The liver cell cords were arranged radially around the central vein, and there was no pathological change. The spleen tissue had a classic morphological form, with a distinct and unambiguous boundary of the white pulp area. The lymphocytes in the lymphatic sheath surrounding the central artery were tightly packed together. The lung tissue had a fully developed morphological structure, yet, the alveolar wall of each group showed increased thickness. The thickening of the alveolar wall in the D, PC, and NC groups was more serious, which was considered to be related to cough and asthma in clinical practice. The morphological structure of kidney tissue of weaned piglets in each group was normal and clear, the arrangement of renal tubules and glomeruli was regular, and the space, volume and glomerular volume of renal capsule were within the normal limits. The results of small intestinal tissue sections in each group showed that the morphological structure of the duodenum, jejunum, and ileum was normal and complete, and the intestinal villi were compact and complete without pathological changes (Figure 3).

### 3.10 Effects of matrine on intestinal flora of weaned piglets

**Figure 4:**
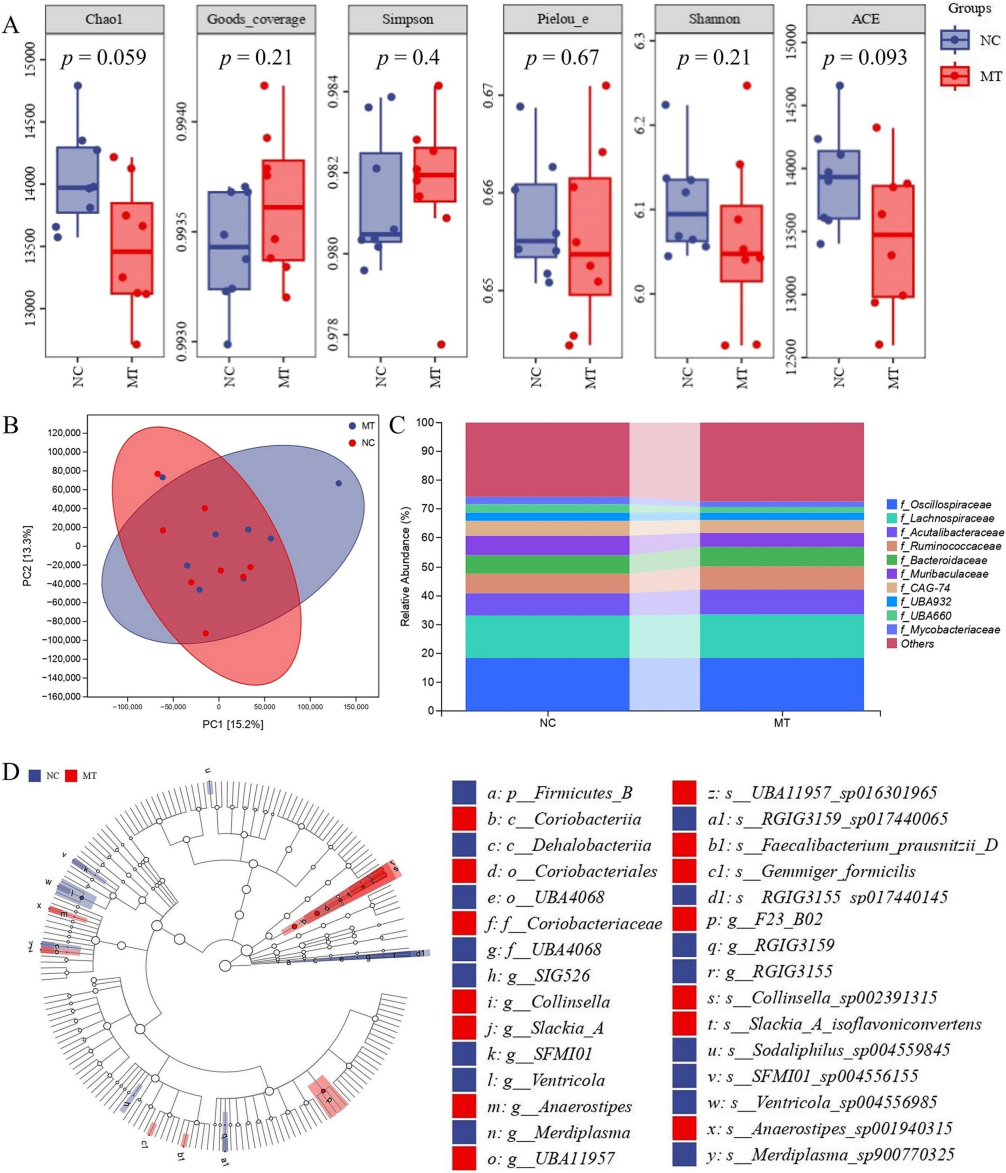
The results of fecal metagenomics in weaned piglets. **(A)** α diversity analysis. **(B)** PCA analysis. **(C)** Species composition analysis. **(D)** LEfSe analysis.

The results of metagenomics of piglet feces showed that there was no significant difference in Chao1, Goods coverage, Simpson, Pielou e, Shannon, and ACE index between the 0.75 mg/kg matrine group (MT) and NC group (Figure 4A), indicating that matrine had no significant effect on the species richness, evenness and diversity of intestinal flora in piglets. The results of PCA analysis showed that the samples of the NC group and MT group were not completely separated into two clusters (Figure 4B), this suggests that matrine had no significant effect on the composition of intestinal flora in piglets. The results of species classification histogram analysis showed that the species composition of the NC group and the MT group was basically the same at the family level, which was composed of Oscillospiraceae, Acutalibacteraceae, Ruminococcaceae, Muribaculaceae, etc., but some families were different between the two groups. Compared with the NC group, the Ruminococcaceae and Acutalibacteraceae of the MT group showed an increasing trend. Muribaculaceae showed a decreasing trend (Figure 4C). LEfSe analysis showed that at the genus level, compared with the NC group, Collinsella, Slackia A, Anaerostipes, etc. in the MT group were significantly increased, and RGIG3155, Merdiplasma, Ventricola, etc. were significantly decreased in the MT group (LDA > 3, *p* < 0.05). At the species level, compared with the NC group, *Gemmiger formicilis*, *Collinsella sp002391315*, *Slackia A isoflavoniconvertens*, *Faecalibacterium prausnitzii D*, *UBA11957 sp016301965*, and *Anaerostipes sp001940315* were significantly increased in the MT group, while *Ventricola sp004556985*, *RGIG3155 sp017440145*, *Sodaliphilus sp004559845*, *SFMI01 sp004556155*, *Merdiplasma sp900770325*, *RGIG3159 sp017440065* were significantly decreased in the MT group (LDA > 3, *p* < 0.05) (Figure 4D).

### 3.11 Effects of matrine on fecal metabolomics of weaned piglets

**Figure 5:**
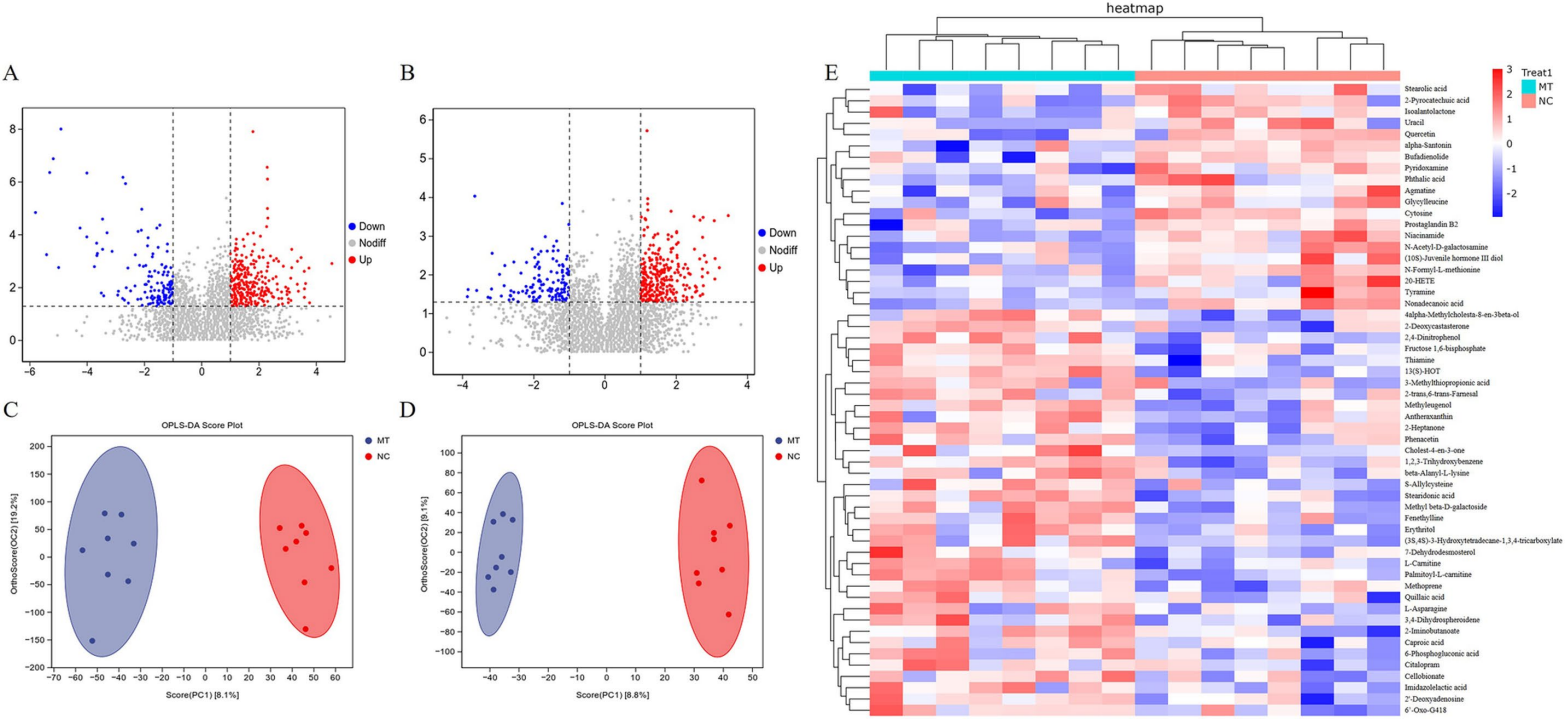
The results of a non-targeted metabolomics study on feces of weaned piglets. **(A)** Volcanic diagram in positive ion mode. **(B)** Volcanic diagram in negative ion mode. **(C)** OPLS-DA analysis in positive ion mode. **(D)** OPLS-DA analysis in negative ion mode. **(E)** Cluster analysis of differential metabolites.

The results of volcano plot analysis revealed that matrine caused significant changes in the content of some metabolites in the feces of weaned piglets (*p* < 0.05) (Figures 5A,B). The results of OPLS-DA analysis showed that the samples in the 0.75 mg/kg matrine group (MT) and NC group were clustered into two clusters, respectively, indicating that there were specific metabolomes in feces under the effect of matrine (Figures 5C,D). The results of cluster analysis of differential metabolites showed that compared with the NC group, 36 metabolites such as 13(S)-HOT, methyl beta-D-galactoside, palmitoyl-L-carnitine, thiamine, 6-Phosphogluconic acid, phenacetin and fructose 1,6-bisphosphate were significantly up-regulated in MT group. Compared with the NC group, 20 metabolites such as niacinamide, cytosine, nonadecanoic acid, pyridoxamine, tyramine, and stearolic acid were significantly down-regulated in the MT group (VIP > 1, *p* < 0.05) (Figure 5E).

### 3.12 Mechanism analysis of matrine improving growth performance of piglets

**Figure 6:**
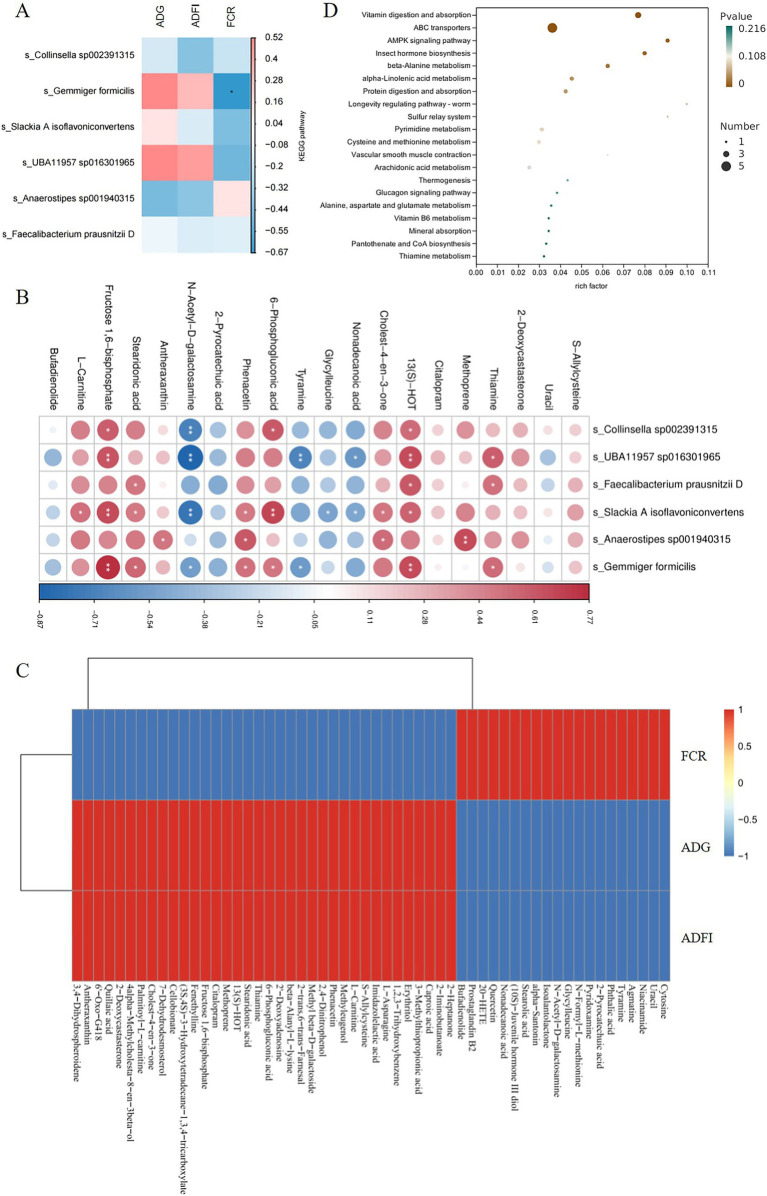
Results of the correlation analysis. **(A)** The results of correlation analysis between metagenomics and production data. **(B)** Correlation analysis between metagenomics and metabolomics. **(C)** The results of correlation analysis between metabolomics and production data. **(D)** Metabolomics KEGG analysis. * indicates *p* < 0.05, and ** indicates *p* < 0.01.

Spearman correlation analysis using the Mothur software was performed to calculate the correlation between fecal metabolomics, metagenomics, and production data. The results of correlation analysis between metagenomics and production data showed that *Gemmiger formicilis* was significantly negatively correlated with FCR (*p* < 0.05), and positively correlated with ADG and ADFI. Therefore, *Gemmiger formicilis* plays an important role in improving ADG, ADFI and reducing FCR in piglets (Figure 6A); The correlation analysis between fecal metabolomics and metagenomics showed that *Gemmiger formicilis* was significantly positively correlated with thiamine, 13 (S)-HOT, 6-phosphogluconic acid, phenacetin, stearidonic acid and fructose 1,6-bisphosphate (*p* < 0.05) (Figure 6B). Furthermore, metabolites such as thiamine, 13 (S)-HOT, 6-phosphogluconic acid, phenacetin, stearidonic acid and fructose 1,6-bisphosphate were positively correlated with ADG and ADFI, and negatively correlated with FCR (Figure 6C). The results of KEGG analysis of fecal metabolomics showed that vitamin digestion and absorption, ABC transporters were the first two pathways with the most significant changes caused by matrine (*p* < 0.01). In the vitamin digestion and absorption pathway, thiamine was significantly up-regulated (*p* < 0.05), niacinamide and pyridoxamine were significantly down-regulated (*p* < 0.05). In the ABC transporters pathway, thiamine, erythritol, 2′ -Deoxyadenosine, methyl beta-D-galactoside were significantly up-regulated (*p* < 0.05), phthalic acid was significantly down-regulated (*p* < 0.05) (Figure 6D). It can be seen that thiamine is a common metabolite in the two metabolic pathways, indicating that the change of thiamine content is the most important effects of matrine on fecal metabolism of weaned piglets. In summary, matrine may play a role in improving the production performance of piglets by affecting *Gemmiger formicilis* and thiamine.

## 4 Discussion

The application of traditional Chinese veterinary medicine has a long history and definitive clinical efficacy, which significantly contributes in the prevention and control of livestock and poultry diseases. National governments and international organizations worldwide are progressively acknowledging and prioritizing the implementation and advancement of traditional Chinese veterinary medicine. More than 40 countries around the world are engaged in research, implementation and promoting traditional Chinese veterinary medicine. Announcement No.194 of the Ministry of Agriculture and Rural Affairs of the People’s Republic of China pointed out that all growth-promoting drug feed additives except traditional Chinese veterinary medicine should be withdrawn. As a feed additive, traditional Chinese veterinary medicine has shown the effects of promoting growth (22), anti-inflammation (23), anti-oxidation (24), and improving immune function (25) in livestock and poultry breeding. At present, ASFV, PCV2, and other viruses have caused significant economic losses to the pig industry in China and the world (26, 27). The extensive use of antibiotics has posed a serious threat to animal health and human health (28). Therefore, it is imperative and urgent to develop safe and effective traditional Chinese veterinary medicine feed additives.

To investigate the effects of matrine on the growth performance of weaned piglets, we first conducted an acute toxicity test in ICR mice following the “Guidelines for Acute Toxicity Test of Veterinary Drugs (LD_50_ Determination).” The results revealed an oral LD_50_ of 202.54 mg/kg for matrine. Guided by the “Technical Guidelines for the Safety and Effectiveness of Growth-Promoting Veterinary Chinese Medicines” and “Guidelines for 30- and 90-days feeding trials of veterinary drugs,” a 180-day rat feeding trial was conducted at 24.5, 35, and 50 mg/kg matrine (25% of LD_50_), demonstrating no adverse effects on hematology, serum biochemistry, and organs morphology. Using body surface area conversion, these doses correspond to 5.44 ~ 11.11 mg/kg in piglets, which are commercially impractical due to prohibitive costs. To establish a feasible dose range, we referenced Boluohui San, a registered alkaloid feed additive containing 1.5% sanguinarine (effective dose: 0.75 mg/kg). Aligning with this benchmark, we tested matrine at 0.375 ~ 3.000 mg/kg to balance efficacy (growth performance) and cost-effectiveness, ensuring translational relevance for industrial applications.

The blood biochemical test results of weaned piglets showed that matrine had no significant effect on the biochemical parameters. Specifically, hepatic parameters (e.g., ALB, ALT) and renal indicators (e.g., BUN, Cr) remained within reference ranges, suggesting that dietary supplementation with 0.375 ~ 3 mg/kg matrine did not induce liver and kidney toxicity in weaned piglets. The findings of morphological characteristics of several organs showed that there were no pathological changes in the heart, liver, spleen, kidney, and small intestine of weaned piglets in each group. Therefore, it is indicated that 0.375, 0.75, 1.5, and 3 mg/kg matrine can be safely used in weaned piglets. Growth performance tests demonstrated that dietary supplementation with 0.375, 0.75, and 1.5 mg/kg matrine for 34 days improved the ADG of weaned piglets, with 0.75 and 1.5 mg/kg showing superior efficacy in reducing the FCR. Notably, the lack of efficacy observed in the 3 mg/kg matrine group may reflect a threshold effect, whereby excessive matrine could suppress beneficial microbial activity or induce mild metabolic stress, as evidenced by its limited capacity to alleviate diarrhea. This parallels findings from studies on fermented ginseng in alleviating antibiotic-associated diarrhea in rats, where high doses similarly compromised therapeutic outcomes due to intestinal flora (29). PCV2 vaccine antibody positivity rate analysis revealed that the 0.75 mg/kg matrine dose exhibited the strongest enhancement effect on PCV2 vaccine antibody seropositivity. Additionally, during the trial period, we observed that supplementation with 0.75 mg/kg matrine alleviated diarrhea in piglets subjected to feed transition-induced digestive stress, indicating its potential to mitigate dietary adaptation challenges. Consequently, we further investigated its effects on fecal metagenomics and untargeted metabolomics in weaned piglets. The potential mechanisms underlying matrine’s effects on piglet growth performance were explored through integrated analysis, including metagenomic profiling, metabolomic characterization, and their correlations with production parameters.

The results of metagenomics analysis showed that 0.75 mg/kg matrine increased the abundance of *Gemmiger formicilis*, *Collinsella sp002391315*, *Slackia A isoflavoniconvertens*, *Faecalibacterium prausnitzii D*, *UBA11957 sp016301965*, and *Anaerostipes sp001940315* in the intestine of weaned piglets. At the same time, metabolites such as 13(S)-HOT, methyl beta-D-galactoside and thiamine were significantly increased in 0.75 mg/kg matrine group. Correlation analysis showed that *Gemmiger formicilis* was positively correlated with ADFI, ADG, 13(S)-HOT, phosphogluconic acid, fructose 1,6-bisphosphate, stearidonic acid, phenacetin, and thiamine, and significantly negatively correlated with FCR. In addition, these mentioned metabolites were positively correlated with ADG and ADFI, and negatively correlated with FCR. Through KEGG analysis of fecal metabolomics, we further found that thiamine was a common metabolite in vitamin digestion and absorption and ABC transporter pathway, which were the first two pathways with the most significant changes caused by matrine. In addition, matrine increased the contents of 6-phosphogluconic acid and fructose 1,6-bisphosphate which were glucose metabolites. It is worth noting that thiamine is an important coenzyme in the process of glucose metabolism (30). These findings demonstrate that the most pronounced fecal metabolic alteration induced by matrine was its modulation of thiamine content, while further suggesting its capacity to activate systemic glucose metabolic pathways in weaned piglets. Furthermore, it has been reported that dietary supplementation of thiamine and pyridoxine-loaded vanillic acid-grafted chitosan enhanced the growth performance in the animal model (31). The low abundance of *Gemmiger formicilis* was associated with poor body mass index (BMI) (32). It is suggested that the improvement of piglet production performance is related to *Gemmiger formicilis* and thiamine. In summary, the addition of 0.75 mg/kg matrine to the feed can improve the growth performance of weaned piglets. This growth-promoting effect may be related to the changes in *Gemmiger formicilis* and thiamine.

At present, PCV2, ASFV, and other animal viruses have not been well controlled which seriously affects the healthy development of the pig breeding industry in China (26, 27). In addition, in the actual production process, the farm should give different types of feed to piglets at different growth stages to improve the production efficiency of piglets. Therefore, the actual production process is complex. In order to systematically evaluate the role of matrine in actual production practice, this experiment carried out experimental animal feeding, immunization, and diet replacement according to the piglet feeding plan of Huanshan Pig Farm in Weihai City, Shandong Province, to evaluate whether matrine can improve the production performance and immune function of piglets in the actual production process. During the experiment, it was found that the replacement of feed on the 20th day of the experiment led to clinical symptoms such as diarrhea in piglets but the diarrhea of piglets with 0.75 mg/kg matrine in the feed was reduced. In addition, on the 20th day of administration, all piglets were immunized with the PCV2 vaccine in the pig farm. A large number of studies have shown that traditional Chinese medicine feed additives can enhance the immune effect (33, 34). Therefore, 24 h after the last administration of matrine, the effect of matrine on the positive rate of PCV2 antibody in piglets was investigated. The results showed that compared with the NC group, the positive rate of serum PCV2 antibody in piglets increased by 27 and 33.9%, respectively, after adding 0.375 and 0.75 mg/kg matrine to the feed. However, analyses of serum immune parameters revealed matrine supplementation induced no significant alterations in systemic immune profiles, indicating that matrine selectively enhanced vaccine-specific antibody responses without triggering systemic inflammation or broad-spectrum immune activation. This dual effect of “localized potentiation-systemic stability” suggests that matrine precisely boosts vaccine efficacy while avoiding metabolic burden or tissue injury associated with generalized immune stimulation. Nevertheless, the precise immunomodulatory mechanisms governing this targeted response require further mechanistic validation. In summary, The addition of 0.75 mg/kg matrine to the diet could improve the growth performance of piglets, reduce the diarrhea symptoms caused by diet replacement, and increase the positive rate of the PCV2 antibody.

The price of live pigs sold in the Chinese pig breeding network is 20.69 yuan/kg (August 7, 2024). The price of matrine and Boluohui San are 2,500 yuan/kg and 600 yuan/kg, respectively. Compared with the NC group, the profit per kilogram of feed in the B group can be increased by 0.225775 yuan, and the profit per kilogram of feed in the PC group can be increased by 0.07345 yuan, ultimately indicating that matrine can significantly improve the production efficiency of the pig farm and is superior to Boluohui San. Matrine is an alkaloid extracted from *Sophora flavescens* by organic solvents such as ethanol. *Sophora flavescens* is a plant of the genus Sophora of Leguminosae. It has very rich resources in China, and the price is relatively low. It mainly grows in North China, Henan, Zhejiang, Sichuan, Yunnan, and other regions. In summary, matrine has the advantages of abundant resources, low price, and can improve production performance and production efficiency.

## 5 Conclusion

In summary, the addition of 0.75 mg/kg matrine to the feed has the advantages of improving the production performance of piglets, increasing the positive rate of PCV2 vaccine antibody, and improving the production efficiency of pig farms. The mechanism of matrine improving production performance may be related to *Gemmiger formicilis* and thiamine. In addition, matrine has the advantages of abundant resources and low price in China, and it is urgent to be developed as a pig growth-promoting feed additive to contribute to the safe, efficient, and sustainable development of the pig industry in China and the world.

## Data Availability

The data presented in the study are deposited in an online repository. The names of the repository/repositories and accession number(s) can be found below: https://www.ncbi.nlm.nih.gov/bioproject/PRJNA1280286.

## Glossary

PCV2: Porcine circovirus type 2
ASFV: African swine fever virus
PRRSV: Porcine reproductive and respiratory syndrome virus
ADG: Average daily weight gain
ADFI: Average daily feed intake
FCR: Food conversion rate
EMCV: Encephalomyocarditis virus
MT: Matrine
NC: Negative control group
BS: Boluohui San
LD_50_: Median lethal dose
H&E: Hematoxylin and eosin staining
WBC: White blood cell
LYM: Lymphocyte
MON: Monocytes
NEU: Neutrophil
EOS: Eosinophilic granulocyte
BASO: Basophilic granulocyte
ALY: Alien lymphocyte
LIC: Large immature cells
RBC: Red blood cell
HGB: Hemoglobin
HCT: Hematocrit
MCV: Mean corpuscular volume
MCH: Mean corpuscular hemoglobin
MCHC: Mean corpuscular hemoglobin concentration
RDW-CV: Red blood cell distribution width coefficient of variation
PLT: Platelet
MPV: Mean platelet volume
TP: Total protein
ALB: Albumin
GLB: Globularproteins
TBIL: Total bilirubin
ALT: Alanine aminotransferase
AST: Aspartate aminotransferase
ALP: Alkaline phosphatase
TBA: Total bile acid
CK: Creatine kinase
AMS: Amylase
TG: Triglyceride
Chol: Cholesterol
GLU: Glucose
Cr: Creatinine
BUN: Blood urea nitrogen
T-CO2: Total carbon dioxide
Ca: Calcium
P: Phosphorus
